# A CROSS-NATIONAL COMPARISON OF RISK FACTORS ASSOCIATED WITH THE MOTORIC COGNITIVE RISK (MCR) SYNDROME

**DOI:** 10.1093/geroni/igad104.0945

**Published:** 2023-12-21

**Authors:** Oshadi Jayakody, Helena Blumen, Joe Verghese

**Affiliations:** Albert Einstein College of Medicine, Bronx, New York, United States; Albert Einstein College of Medicine, Bronx, New York, United States; Albert Einstein College of Medicine, Bronx, New York, United States

## Abstract

The Motoric Cognitive Risk (MCR) syndrome predicts risk of Alzheimer’s disease and vascular dementia. Cross-national comparisons of risk factors for MCR are important for identifying region-specific factors to design regionally tailored preventive interventions. We examined risk factors associated with MCR in 20,468 adults aged ≥65 years from the U.S Health and Retirement Study (HRS), English Longitudinal Study of Ageing (ELSA), Mexican Health and Aging Study (MHAS), China Health and Retirement Study (CHARLES), Harmonized Diagnostic Assessment of Dementia for the Longitudinal Aging Study in India (LASI-DAD), Survey of Health, Ageing and Retirement in Europe (SHARE) and Brazilian Longitudinal Study of Aging (ELSI-Brazil). MCR was defined as the presence of cognitive complaints and slow gait with no mobility disability and dementia. Associations between demographic [sex, education], medical [hypertension, diabetes, Parkinson’s, heart disease, stroke, obesity, depression, multiple falls], sensorimotor [grip strength, hearing] and behavioral factors [smoking, sedentariness, sleep quality], and prevalent MCR were examined using logistic models. Despite some overlap, cohort-specific risk factors for MCR were identified with relative risk ratios ranging as: 0.4-2.8 for education and multiple falls in HRS, 2.3-7.4 for poor hearing and sedentariness in ELSA, 2.3-2.6 for poor grip and diabetes in MHAS, 1.9-2.2 for depression and sedentariness in CHARLES, 0.5-5.3 for education and stroke in LASI-DAD, 0.3-2.4 for education and depression in SHARE and 2.4-4.0 for multiple falls and stroke in ELSI-Brazil. In this cross-sectional, cross-national comparison of 21 countries, MCR associated risk factors were region-specific, laying the foundation for tailored interventions to prevent MCR and subsequently dementia.

